# Re-Orienting Smartphone-Collected Car Motion Data Using Least-Squares Estimation and Machine Learning

**DOI:** 10.3390/s22041606

**Published:** 2022-02-18

**Authors:** Enrico Bassetti, Alessio Luciani, Emanuele Panizzi

**Affiliations:** Department of Computer Science, Sapienza University of Rome, 00161 Rome, Italy; luciani.1797637@studenti.uniroma1.it (A.L.); panizzi@di.uniroma1.it (E.P.)

**Keywords:** smartphone, parking, sensing, implicit interaction, machine learning, curb, parallel, angle parking, smart city, context aware

## Abstract

Smartphone sensors can collect data in many different contexts. They make it feasible to obtain large amounts of data at little or no cost because most people own mobile phones. In this work, we focus on collecting motion data in the car using a smartphone. Motion sensors, such as accelerometers and gyroscopes, can help obtain information about the vehicle’s dynamics. However, the different positioning of the smartphone in the car leads to difficulty interpreting the sensed data due to an unknown orientation, making the collection useless. Thus, we propose an approach to automatically re-orient smartphone data collected in the car to a standardized orientation (i.e., with zero yaw, roll, and pitch angles with respect to the vehicle). We use a combination of a least-square plane approximation and a Machine Learning model to infer the relative orientation angles. Then we populate rotation matrices and perform the data rotation. We trained the model by collecting data using a vehicle physics simulator.

## 1. Introduction

The widespread presence of powerful smartphones in people’s pockets incentivized discovering new ways to exploit their sensors. One setting in which smartphone sensors can be beneficial is cars: innovative transportation challenges need vehicle data, and accelerometers and gyroscopes in users’ smartphones can replace more sophisticated and expensive approaches for motion data collection. Using a tool that is so popular among people makes data collection much more scalable.

Unfortunately, obtaining high-quality motion data from crowdsensing is far from being straightforward. Users can position their smartphone in the car in many different ways (e.g., the smartphone can be in a pocket with the screen facing the car window or in a cup holder facing the driver), and often they use it and move it while traveling. These facts can impact the interpretation of the sensed data due to an unknown orientation, making the collection useless.

Therefore, this work aims to automatically orient the data collected along the car’s axes, whatever the orientation of the smartphone in the car. We accomplish this by performing a series of data point rotations in a 3D space and similar operations, using a Machine Learning model to detect the rotation angle. We trained the model with data obtained by a vehicle simulator to speed up the collection procedure, although the simulation has some drawbacks, as described below.

**Figure 1:**
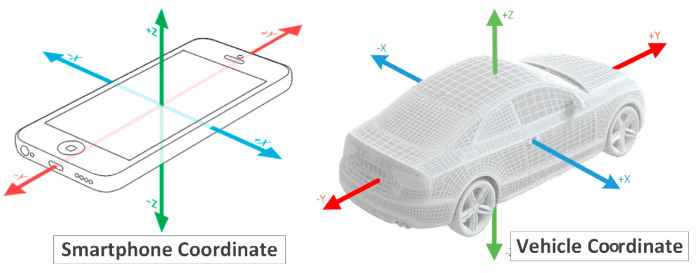
Smartphone and car axes resulting orientation.

The resulting processed data looks as if collected by a smartphone placed horizontally in the car, with the screen facing the car roof and the top oriented towards the car’s front (Figure 1).

### Overview

First, we will discuss finding the horizontal least-squares plane and the subsequent data rotation. The least-square plane allows the understanding of the orientation of Euler angles between the raw data points and the gravity plane. Then, it is possible to rotate the smartphone’s data according to those two angles: pitch and roll. The following step is to find the remaining orientation yaw angle. We use an ML technique to find it depending on the car’s motion data points. The final sections will cover all the processes that took us to develop the model and perform the eventual yaw rotation: data collection in the simulation, model training, data rotation, and performance evaluation.

## 2. Related Work

Detecting the smartphone orientation is a problem in many areas when using crowdsensing or unsupervised experiments. Approaches and techniques described in the literature differ according to the needs and goals of the experiment at hand: some situations require absolute and accurate orientation; in other cases, an orientation ‘class’ is sufficient; others tackle the problem by orienting the smartphone in a particular situation and infer different situations when the orientation changes. Not all works rely on machine learning. The advantages of some approaches are related to computational complexity and device consumption. Drawbacks are mainly related to the accuracy of results and the lack of generality.

Works regarding the orientation of a smartphone in a car are available in the literature. Some of them are about classifying the type of placement of a smartphone in the vehicle by devising several positions that it could take: [1] describes a clustering approach to learn different classes of smartphone positions. However, there is no focus on processing the data to standardize it to a fixed position and orientation. Our work, instead, mainly focuses on that part. Another study, [2], focuses on detecting front-to-back and side-to-side car moving dynamics to understand whether the driver is using the smartphone. Our technique to infer the smartphone’s orientation is similar to this approach, although it aims at a different purpose. In [3] machine learning is used to infer the entire 3D rotation matrix. Instead, we first make a horizontal alignment with a more deterministic least-square approximation and then use ML to infer the remaining 1-axis angle, obtaining more accurate results. Meanwhile, [4] presents a way to infer smartphone-vehicle alignment based on GNSS and inertial measurements. The proposed solution requires an always-on GNSS receiver and accelerometer data from the smartphone, while our approach uses the accelerometer and the gyroscope.

The sensor orientation problem is also tackled in other areas to cope with sensing data collected using smartphones or smartwatches in non-controlled environments. For example, we report some Human Activity Recognition (HAR) works and building structural monitoring research. As reported in [5], many HAR works need to assess the smartphone orientation with respect to the body in free-living experiments. Del Rosario et al. [6] learn the Inertial Measurement Unit orientation during walking to acquire a reference orientation against which sitting or lying can be inferred. Straczkiewicz et al. [7] use accelerometric data to assess location, placement and orientation of a body-worn smartwatch in unsupervised experiments. They rely on the median acceleration on the three axes, expecting different signs of the gravitational vector to identify one out of eight different smartwatch positions.

Several works related to structural monitor of buildings using smartphones need to solve the smartphone orientation problem, such as: [8,9,10,11]. Kong et al. [8] determine phone orientation based on known modal frequencies and corresponding mode shape of the building, finding the appropriate rotation to reproduce motion. With respect to our work, Kong et al. rely on ground truth data from external sources regarding the solicitation of the building. Ozer et al. [11] perform a coordinate system transformation using magnetic north and gravitational direction as reference vectors and compare them with the structure’s main directions. Their approach is the most similar to ours, although they rely on absolute acceleration data and magnetometer, accelerometer and gyroscope sensors, while our proposal only relies on accelerometers and gyroscopes. Furthermore, since we deal with vehicles in continuous movement and not still buildings, the idea of using the magnetic north as an input is not easily applicable in our case. So, we instead use ML to make sense of data coming from the other sensors.

Finally, there are works in the literature that exclude the orientation calculation by simply fixing the device in a given position [12] and are thus not comparable to our work.

## 3. Materials and Methods

### 3.1. Data Collection

We developed a smartphone app for iPhone that collects sensor data during a car trip. Although we collected data using an iPhone, we do not leverage any specific characteristic or feature; we expect to have similar performance on other devices which carry the same family of sensors, such as Android smartphones.

The Bluetooth connection of the smartphone with the car IVIS triggers the app, and since that moment, it samples data points at a 10 Hz rate till the Bluetooth disconnection. Each sample includes rotation (from the gyroscope) and acceleration. Rotation and acceleration are on the three axes *X*, *Y* and *Z*.

### 3.2. Smartphone Motion Sensors Data Pre-Processing

In this paragraph, we observe in depth the required preliminary data processing steps when collecting data in a real-world scenario and employing smartphone motion sensors. In this case, many external factors influence the data samples, and, therefore, we need to perform some actions to clean the data and prepare it for the following processing steps.

Every time a person moves the smartphone, it changes position and orientation with respect to the car. We need to discard all the data values related to the manual movements of the smartphone and split the trip into intervals in which the phone is stable in an orientation relative to the car so that we can detect it and reorient all the samples along the car axes.

We first use gyroscope samples to detect if the smartphone is idle in the car or is in the user’s hands. We could confirm the observation in [1] that the smartphone’s rotation rate is relatively stable when positioned in the car, while it peaks when the smartphone is manually moved (for example, when the driver gets out of the car). Doing some tests, we established a threshold of 85 deg/s and associated more significant rotation rates with manual movements of the smartphone.

Therefore, we use gyroscope peaks to split the trip into intervals, in each of which the smartphone has a constant orientation.

### 3.3. Least Squares

**Figure 2:**
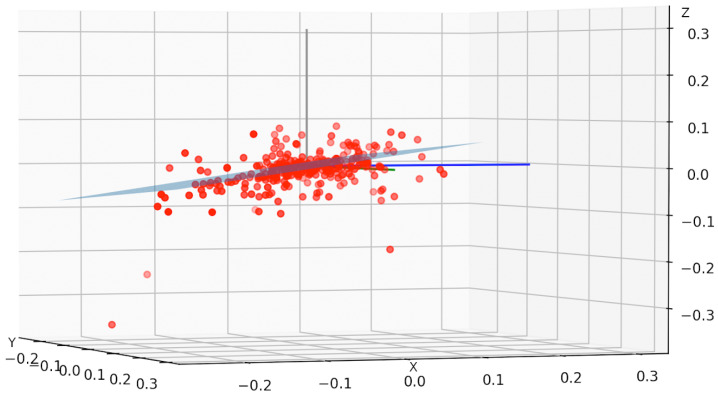
3D distribution of acceleration points. The least squares plane is drawn in blue (side view)—values are in ${m/s}^{2}$.

The acceleration samples are studied to figure out which is the most probable car horizontal plane (Figure 2). During a car trip, accelerations on the vertical axis (i.e., *Z*-axis) are close to 0 since they are due mainly to road disturbances and beginnings of ascents or descents. So, in most cases, the accelerations are concentrated on the axes *X* and *Y*. This fact lets us compute the least-squares plane [13] for the acceleration points in time.

**Figure 3:**
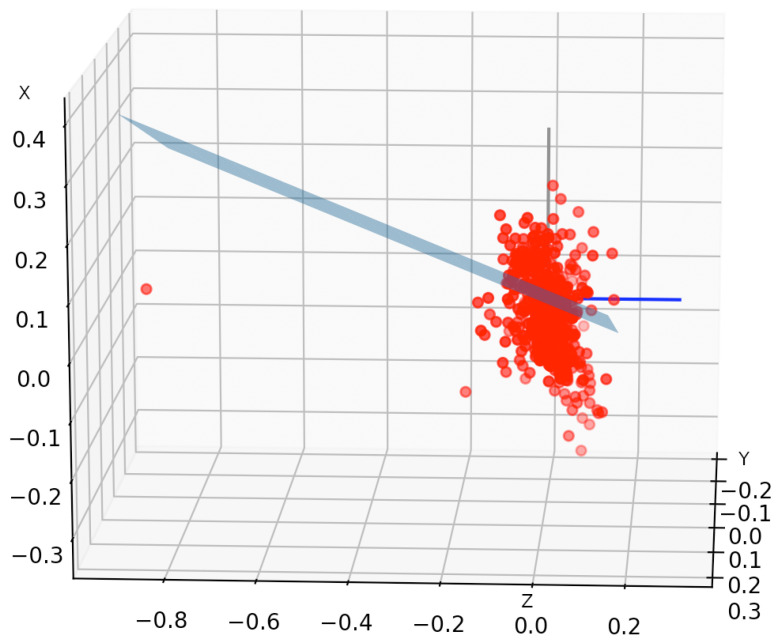
Outliers removal from 3D distribution of acceleration points—values are in ${m/s}^{2}$.

However, before deriving least-squares plane candidates, we need to remove outliers (Figure 3). To do that, we use the Z-Score algorithm [14] (indicated as β). First, we compute the standard deviation for every axis. Then, we empirically set the max value that we accept for Z-Score β to 5 (value chosen by optimizing FAR and FRR when looking for points of the plane in our dataset), and calculate the Z-Score $\beta_{p_{i}}$ as in Equations (1)–(3) for each point $p_{i}$ and for axis *x*, *y* and *z*.

(1) $$\beta_{p_{i}}^{x}=\frac{\left(x_{p_{i}}-\mu^{x}\right)}{\sigma^{x}}$$

(2) $$\beta_{p_{i}}^{y}=\frac{\left(y_{p_{i}}-\mu^{y}\right)}{\sigma^{y}}$$

(3) $$\beta_{p_{i}}^{z}=\frac{\left(z_{p_{i}}-\mu^{z}\right)}{\sigma^{z}}$$

Finally, we discard all points exceeding the max β value on any axis. This removal is only temporary to compute the right least-squares plane. Once the plane coefficients are defined, we use them to rotate all points in the space towards the horizontal plane (i.e., the plane Z=0).

The idea behind the rotation is to rotate each acceleration data point (i.e., a 3D vector composed of the acceleration components on the three axes: *X*, *Y* and *Z*) around the axis common to the two planes.

Let *N* be the vector normal to the plane Z=0, *M* be the vector normal to the newly found car horizontal plane, dot() the dot product calculation, norm() the normalization and cross() the cross product calculation. First, we calculate the required angle Θ as the ratio between the dot product of the plane vector *N* and the car horizontal plane *M*, and the product of their normalization (Equation (4))

(4) $$cos\Theta =\frac{dot(M,N)}{\left(norm(M)*norm(N)\right)}$$

Then, the axis around which the rotation will be made is calculated as the ratio between the cross product of *N* and *M* and the normalization of their cross product (Equation (5))

(5) $$\frac{cross(N,M)}{norm(cross(N,M))}$$

Finally the rotation matrix is calculated as:

(6) c=cosΘ

(7) $$s=\sqrt[{2}]{[}1-cc]$$

(8) C=1−c

(9) $$Q\left(\Theta \right)=[\begin{array}{ccc} xxC+c & xyC-zs & xzC+ys\\ yxC+zs & yyC+c & yzC-xs\\ zxC-ys & zyC+xs & zzC+c \end{array}]$$

The rotation matrix calculation is further described in [15].

Then, the *Q* matrix can be used to rotate every single point $p_{i}$ as

(10) $$p_{i}^{\prime }=dot\left(Q,p_{i}\right)$$

If the two planes are similarly oriented, the rotation does not happen since it would not improve the precision. At this point, the processed data has a similar shape to the one that a smartphone would collect in a parallel position relative to the car’s horizontal plane.

### 3.4. Data Collection in the Simulation

Collecting a sufficient amount of data in actual vehicles is a non-trivial task for different reasons. Since the considered system deals with supervised learning and requires labeled data, the collection process has to be conducted exclusively by operators that are well aware of the system requirements and pay close attention to the collection details. Wrongly collected or incorrectly classified data are deleterious for the quality of the final model. Moreover, operators should annotate details about the smartphone’s orientation in the car during the collection and input them into the dataset. Measuring orientation angles in a natural environment can be challenging for human operators. Finally, to have sufficient samples, the operators would have to make many authentic vehicle trips, which is time- and fuel-consuming. We estimate that a satisfactory dataset should contain a few thousand samples. Random forests perform relatively well on datasets of this size.

**Figure 4:**
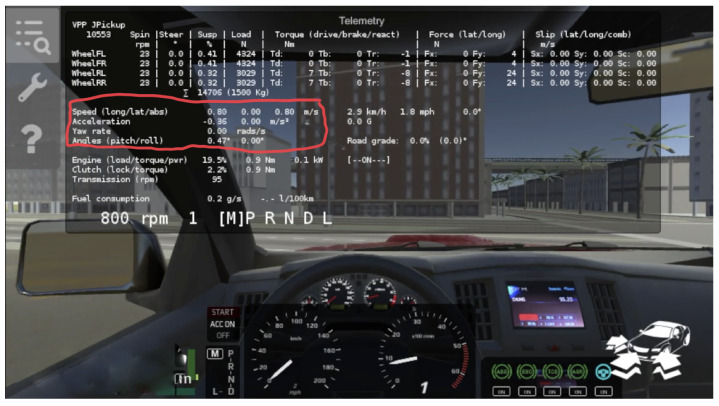
Vehicle Physics simulator. In the red box, the data that we collected during the simulation.

The simulation provided by vehiclephysics.com (Figure 4) allows us to emulate the behavior of a car that moves almost the same as a real one in the real world. The simulation generates all the movements and data we need from the car. We can extrapolate these data and prepare them the same way we do in the actual samplings.

#### 3.4.1. Structure of the Simulation

The simulation comes in a customizable Unity 3D project, adaptable to specific needs. As a foundation, there are some basic vehicles and maps provided. One of the default maps includes on-street parking with lines drawn on the floor that specify the exact positions of the parking lots. This map also has city streets and highways to emulate real driving situations and different parking motions.

The two default car models provided are a sports car and a pickup truck. We decided to use the latter because its acceleration and brakes are more balanced and similar to most cars. Furthermore, we decided to limit the vehicle’s maximum acceleration and braking power to avoid unnatural levels. In fact, by default, the vehicle would accelerate significantly before automatically shifting the gear. In that case, the engine revolutions indicator would touch 4000–5000 RPM, and the acceleration would be too disruptive. These levels are far from usual city driving.

The vehicle can be controlled with a keyboard or a game controller. The main difference between the two options is that the car control is discrete in the first case, while in the second one, it is continuous thanks to moving cursors. Thus we can apply a variable acceleration to the vehicle that better resembles a real interaction with the car.

Since this is a Unity 3D project, we can also access external frameworks. We experimented with connecting the simulation to an Oculus VR. The Unity project sets a camera in the car by default. In order to connect the Oculus visor, we had to replace the default camera with the Oculus one and attach it to the vehicle. After doing that, the connection was seamless, and the driving experience was even more realistic and straightforward to simulate.

#### 3.4.2. Obtaining the Required Simulation Data

The Vehicle Physics Pro simulation offers a comprehensive API and access to vehicle components. In particular, there is a wide variety of metrics related to physics values in a whole section of the API dedicated to telemetry data. The telemetry section is responsible for collecting the physics data generated by the core simulator and making them programmatically readable.

The Vehicle Physics Pro simulator is written in C#, and its code is accessible from third-party scripts, such as ours. We used a “vehicle” object that contains most of the information related to the car’s motion. This way, we extracted all the features that we needed:

- acceleration on *X*, *Y* and *Z* axes (represented as the local acceleration);
- rotation rate on *X*, *Y* and *Z* axes (represented as the angular velocity);
- speed.

The script launches when the simulation starts, and it contains a thread that can be in two states: “not recording” and “recording”. As soon as the script executes, it spawns the new thread in the “not recording” state. When the user presses a specific key on the keyboard, the thread goes into the “recording” state and starts sampling the data at a fixed rate (10 times per second). After the pressure of another button, the thread goes back to the “not recording” state and saves the samples in a JSON file. So, we followed this procedure: (i) starting the simulation; (ii) pressing the record button; (iii) driving the car around the map; and (iv) stopping the recording. We did this many times, trying to cover as much terrain as possible. We tried to make each drive slightly different from the previous ones to populate the dataset with diverse information. We chose different streets, turns, acceleration and braking patterns to do this. We also simulated parking motions in different styles and shapes, such as parallel parking along the street and angle parking in the dedicated parking location.

#### 3.4.3. Simulated Data Processing

Unlike the actual data situation, simulated data do not have human interference, so the gyroscope fluctuations problem does not appear. The data collected from the simulator can be seen as coming from a “virtual smartphone” positioned parallel to the car’s horizontal plane and heading towards its front.

#### 3.4.4. Features Extraction for Orientation Model

The required angle in the simulated data is 0°. So, to create some valid labels, the data can be rotated on the horizontal plane of a random angle. This kind of 3D rotation is based on Euler angles [16]. We labeled the sample with the random angle value. We repeated this process many times for every sample, picking random angles, eventually obtaining a larger dataset. In our case, we repeated it 100 times. Starting from a 150-sample dataset, we rapidly ramped up to a more significant 15,000-sample one.

We rotated the data points around the Z-axis (i.e., the axis orthogonal to the car’s horizontal plane) using a simple rotation matrix (Equation (11), where θ is the randomly picked angle) since it only interests one axis.

(11) $$R_{z}\left(\Theta \right)=[\begin{array}{ccc} cos(\Theta ) & -sin(\Theta ) & 0\\ sin(\Theta ) & cos(\Theta ) & 0\\ 0 & 0 & 1 \end{array}]$$

At this point, we can rotate every single point using the *R* matrix, using the procedure described in [17]:

(12) $$p_{i}^{\prime }=dot\left(R,p_{i}\right)$$

where dot() is the dot product calculation.

At this point, we must compose the feature vector. We used the following features for each sample: acceleration mean for *X* and *Y* axes; acceleration standard deviation for *X* and *Y* axes; acceleration mean error for *X* and *Y* axes; rotation rate mean for *X* and *Y* axes; rotation rate standard deviation for *X* and *Y* axes; rotation rate mean error for *X* and *Y* axes. The *Z* axis is not significant in this case.

A tabular dataset is generated, composed of rows of features and a final target value. The file format is CSV (comma-separated values), so the first row indicates the names of the columns, separated by commas. All the other rows contain data, namely the relative value for every column, including the target value in the target column. Additionally, the data values are separated by commas, according to the column names.

### 3.5. ML Orientation Model

**Figure 5:**
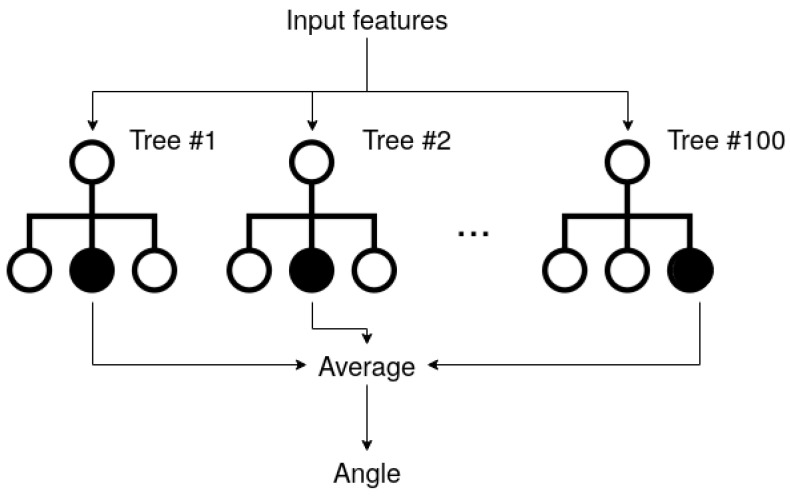
Random forest structure.

We created the model using Apple’s Create ML tree ensemble regressor, a predictive model composed of a weighted combination of multiple regression trees, also called random forest [18] (Figure 5).

A decision tree regressor is a predictive model that uses a set of binary rules to calculate a target value. Each tree is a simple model with branches, nodes and leaves. Every decision is made discerning on a feature of the initial set and is structured as a node that branches out in two separate nodes at the lower layer of the tree. Finally, leaves are the target values. A random forest uses a collection of different trees with random constraints limiting their learning freedom to reduce variance for the overall model. Some examples of constraints applied to the creation of models are maximum depth, the maximum number of features, and the minimum number of samples to do a split.

Create ML offers a wide range of tools to create, train and test new ML classifiers and regressors for different purposes, either via a GUI or programmatically in Swift language. We need a regressor that outputs a floating-point number based on the input sample for our purposes. In our case, we need to obtain an angle value as the output, expressed in radians, from 0 up to 2π.

**Figure 6:**
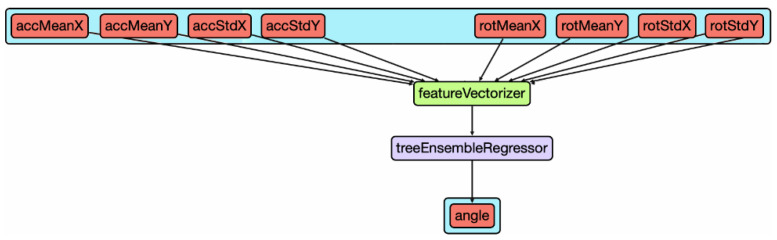
Orientation ML model structure.

This kind of model receives a vector of features as input (Figure 6). In particular, the input features of the model are the mean accelerations, mean rotation rates, acceleration’s standard deviations, and rotation rate’s standard deviations. These features have been chosen because they contains information about car maneuvers and movements, and we believe that these are good descriptors for understanding the angle between the smartphone and the car headings. They are taken along the X and Y axes, excluding the Z axis. We exclude the Z axis because the data is oriented in the horizontal plane at this point in the process. Therefore, only road bumps and slope changes are caught along that axis. That kind of information is of no help for estimating the yaw angle. These features are computed over the entire collection segment. We do so to compress data coming from the entire interval into a single group of features that is invariant to the length of the segment and can be processed through a single pass of the model.

The set of features used as input are previously vectorized and made compatible with the model. The initial set of features also contained mean error values. However, after performing a feature-importance analysis (Table 1), we discovered that they were of no help for the model and excluded them. Interestingly, this analysis discovered that the feature representing the mean of the rotation rate along the Y-axis takes up almost 59% of the total importance.

The training procedure does not imply long waiting times since this model does not have the recursive complexity and layers depth of a more sophisticated neural network.

## 4. Results

### Orientation Model Testing

By definition, we collected correctly oriented data in the simulation with our virtual smartphone in the car. So, we could take advantage of this aspect and generate samples by taking simulation recordings and rotating the motion data of a chosen yaw angle. Since we could choose this angle, we could then use it as our ground truth label during training. In other words, our training set is composed of pairs of purposely rotated car motion recordings as input and their respective angle of rotation as output. Moreover, since we could choose random angles for this preliminary rotation, every recording could be used more than once by rotating it using different angles.

We trained the model on 150 samples (training set) and we tested using 10 recordings (test set). Data have been augmented due to the small size of the dataset: we generated new samples by rotating each recording in 100 random angles. The feature set that reached the best results was composed of the following features for the X and Y axes: acceleration mean; acceleration standard deviation; acceleration error; rotation mean; rotation standard deviation; rotation error. After training the model, we tested it on unseen data, predicting angles. We achieved a root mean square error of 0.34 radians, which means that the model was, on average, only wrong by about 19 degrees. We also computed an R2 score of 0.87. The best possible R2 score would be 1.0. All the results are conveniently reported in Table 2. Since we were performing a regression task, there are no accuracy metrics, but we reported regression metrics.

## 5. Discussion

### 5.1. Applications

The approach described in this paper can be applied to many real-world scenarios where data collected via a smartphone is used to extract any information related to the context of a car or other type of vehicles. For example, the presented method can help understand the current position of an airplane from the smartphone to control the “Airplane mode” switch on departure and landing. Another example can be detecting rolling and yawing in motorcycles to detect crashes or the driver’s erratic behavior.

One use case is the one reported in [19] to classify the parking type of on-street parking lots by analyzing motion data collected via the driver’s smartphone. Accelerometer and gyroscope data points are processed through the technique explained in this article before being fed into the parking type classification model.

### 5.2. Limitations

Limitations of this paper include using simulated data for training and evaluation. However accurate the chosen simulator may be, simulated data cannot follow the same distribution of data collected in real cars. While not influencing the computation of the least-squares plane, this loss of accuracy has a most concerning effect on the training of the ML model. The fact that the dataset only contains simulated data generates a strong bias that makes a potential implementation of the model in a real-world scenario a sub-optimal solution.

MEMS sensors are subjected to various degrees of errors depending on the environment. For example, MEMS gyroscopes have been found to add errors in readings when the temperature changes due to their thermal effect on the silicon chip [20,21]. Our work does not take into account these differences due to the nature of the dataset.

Road surface anomalies (such as potholes or ruts) can introduce noise in smartphone orientation detection. If these anomalies are marginal in the point cloud, they are excluded by the least-squares plane calculation thanks to the outlier cleaning Section 3.3. However, we do not take into account these anomalies in this work.

### 5.3. Ongoing Work

We are going to extend the data collection to a real-world environment using a smartphone in a vehicle, and we will be able to measure the impact of temperature, humidity and road surface anomalies on our work.

## 6. Conclusions

We addressed the problem of reorienting car motion data collected using smartphone sensors, whatever the position and orientation of the smartphone in the car. We could obtain this result using a combination of least square plane and a tree ensemble ML model. The described procedure unrolled in two steps: first, we computed the least-squares plane formed by the acceleration data points, then, using it, we extracted pitch and roll angles by comparing the vehicle’s horizontal plane with the smartphone’s horizontal plane; in the following step, we trained an ensemble ML model that uses regression to infer the difference between the heading of the vehicle and that of the smartphone. This information is the required yaw angle. As described, we used the three angles sequentially to perform the data-point rotations.

## Figures and Tables

**Table 1:** Table of the feature importances.

Feature	Importance (0–1)
accMeanX	0.10108176
accMeanY	0.17573071
accStdX	0.00604311
accStdY	0.0041152
accErrX	0.0
accErrY	0.0
rotMeanX	0.11514755
rotMeanY	0.58944343
rotStdX	0.00560081
rotStdY	0.00283743
rotErrX	0.0
rotErrY	0.0

**Table 2:** Table of the ML model results.

Metric	Value
RMSE	0.34 rad
R2	0.87
Angle Error	19°

## Data Availability

The data presented in this study are openly available in FigShare at https://doi.org/10.6084/m9.figshare.19189421.v1 (accessed on 31 December 2021).

## Abbreviations

The following abbreviations are used in this manuscript:

API	Application programming interface
CSV	Comma-separated value
RPM	Revolutions per minute
JSON	JavaScript Object Notation
FAR	False Acceptance Rate
FRR	False Rejection Rate
ML	Machine learning
